# Synthesis and bioevaluation of novel steroidal isatin conjugates derived from epiandrosterone/androsterone

**DOI:** 10.1080/14756366.2019.1659790

**Published:** 2019-09-02

**Authors:** Shaoyong Ke, Zhigang Zhang, Manli Liu, Wei Fang, Daye Huang, Zhongyi Wan, Ronghua Zhou, Kaimei Wang, Liqiao Shi

**Affiliations:** National Biopesticide Engineering Research Centre, Hubei Biopesticide Engineering Research Centre, Hubei Academy of Agricultural Sciences, Wuhan, China

**Keywords:** Epiandrosterone, androsterone, isatin, conjugate, synthesis, cytotoxic activity

## Abstract

Steroids are classes of natural products widely distributed in nature, which have been demonstrated to exhibit broad biological functions, and have also attracted increasing interest from bioorganic and pharmaceutical researches. In order to develop novel chemical entities as potential cytotoxic agents, a series of steroidal isatin conjugations derived from epiandrosterone and androsterone were efficiently prepared and characterized, and all these obtained compounds were screened for their potential cytotoxic activities. The preliminary bioassay indicated that most of the newly synthesized compounds exhibited good cytotoxic activities against human gastric cancer (SGC-7901), melanoma (A875), and hepatocellular liver carcinoma (HepG2) cell lines compared with 5-fluorouracil (5-FU), which might be considered as promising scaffold for further development of potential anticancer agents.

## Introduction

Cancer is the second cause of death in the world, which denotes a real crisis for public health in the worldwide with the ecological changes and environmental deterioration^1–3^. Although many efforts have been made to control the cancer in the past decades, the outlook is still not optimistic. Among all treatment methods, chemotherapy is still the most common options for cancer patients^2^^,^^4–6^. Therefore, searching for and developing neotype chemical entities with special structure features as potential anticancer agents are an important challenge in the field of drug discovery.

Natural products play important roles in the development of novel drugs^7–11^ because of their unique structures and broad bioactivities, which always offer chemists a range of uncharted chemotypes for the discovery of novel drugs. Steroids are classes of natural products widely distributed in plants, beverages, well-cooked foods and tobacco smoke and with special four-ring structural features, which have been demonstrated to exhibit broad biological functions, and have also attracted increasing interest from bioorganic and pharmaceutical researches^12–14^. On the other hand, isatin is also a natural indole alkaloid consisted in a number of plants, which has also been found to be a common scaffold in various drugs, agrochemicals, and dyes^15–18^.

**Figure 1.**
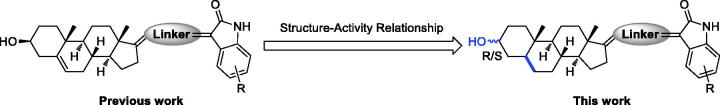
Design strategy of novel steroid-isatin conjugates.

Recently, during the course of our research for highly active compounds based on the scaffold of natural steroids^19–23^, a series of isatin-dehydroepiandrosterone conjugates have been demonstrated to exhibit significant inhibition on the proliferation of human tumor cells^20^, which suggest that this natural four-ring scaffold might contribute to the cytotoxic activity. In order to explore the potential structure-activity relationship, we plan to replace the dehydroepiandrosterone unit with epiandrosterone and androsterone to construct two series of novel isatin derivatives derived from epiandrosterone/androsterone as shown in Figure 1, wishing to identify novel functional molecules with potent antiproliferative effects on tumor cells. The results may provide useful information for design of novel chemotherapeutic drugs.

**Scheme 1.**
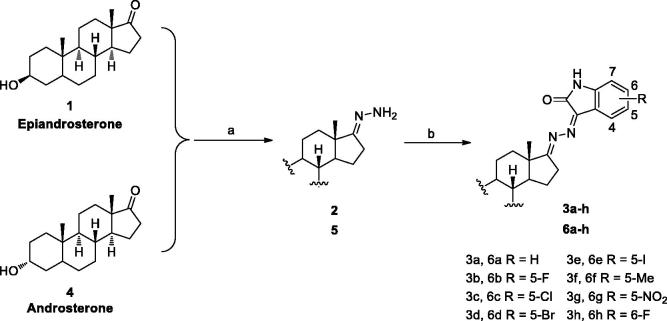
Synthesis of novel steroid-isatin conjugates. Reagents and conditions: (a) NH_2_NH_2_•H_2_O, NH_2_NH_2_•H_2_SO_4_, EtOH/H_2_O, rt; (b) Substituted isatin, EtOH, 30–45 °C.

We utilized epiandrosterone/androsterone as a basic scaffold and planned for the hybridization of the active pharmacophore to the core structure as shown in Figure 1. Therefore, a series of novel steroid-isatin conjugates **3a–h** and **6a–h** were designed and synthesized as shown in Scheme 1, and their potential inhibition on three cell lines (SGC-7901, A875, HepG2) were evaluated by MTT (3–(4,5-dimethylthiazol-2-yl)-2,5-diphenyl tetrazolium bromide) colorimetric method, and the possible structure-activity relationships have also been summarized and discussed.

## Results and discussion

### Synthesis

In the present study, a series of novel steroidal derivatives derived from epiandrosterone and androsterone were efficiently prepared and characterized using a similar method in the reference^20^. The general synthetic route for the synthesis of these steroidal derivatives **3a–h** and **6a–h** is outlined in Scheme 1.

According to the above aforementioned, in order to explore the possible structure-activity relationship, the available diasteromers (epiandrosterone and androsterone) were selected to construct two series of novel steroidal isatin derivatives (**3a–h** and **6a–h**). Firstly, the corresponding epiandrosterone or androsterone was treated with hydrazine hydrate under the catalytic condition of hydrazine sulfate to obtain the intermediates **2** and **5**. Then the compounds **2** or **5** was condensed with various substituted isatin to the target steroidal isatin conjugates **3a–h** or **6a–h**. All the target derivatives were characterized by ^1^H NMR, ^13^C NMR, and ESI-MS spectroscopic analyses.

### Spectroscopy

**Figure 2.**
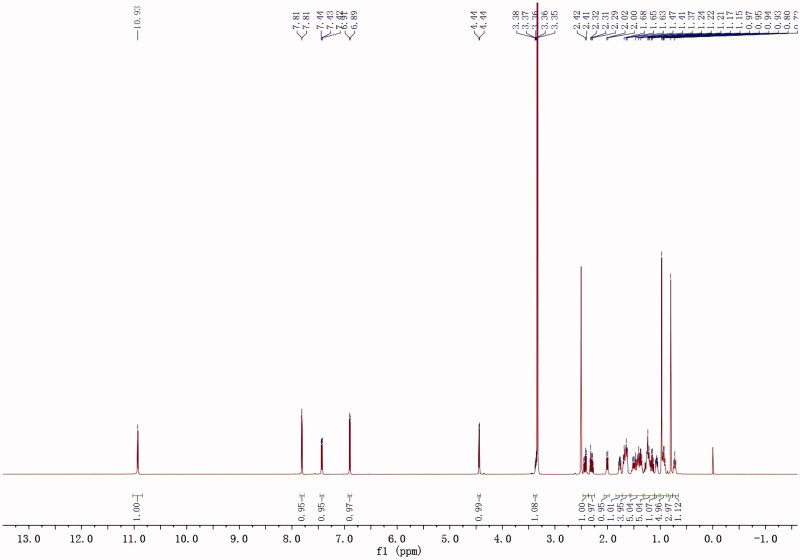
Representative ^1^H NMR spectra analysis of compound **3c**.

All the structures of target derivatives **3a–h** and **6a–h** were confirmed by their ^1^H NMR, ^13^C NMR, and ESI-MS analyses. For ^1^H NMR spectrum of all epiandrosterone or androsterone-isatin derivatives **3a–h** and **6a–h**, the signals at 11.50–10.67 ppm were attributed to NH protons as indicated in molecular structures, and the signals at lower fields in the range of 8.65–6.70 ppm were assigned to the aromatic protons of isatin unit. All ^1^H NMR spectra of target derivatives indicated distinctive signals for protons of hydroxyl group in the part of steroidal ring were resonated as a singlet between *δ* 4.46–4.18 ppm, and the signals of methine proton attached to hydroxyl group were indicated at about 3.30–3.80 ppm. The several set of signals that appeared in their ^1^H NMR spectra at higher fields were attributed to the other protons of steroidal scaffold. All the characteristic peaks observed within the ^1^H NMR spectra for title compounds are given in Experimental Section, and the representative ^1^H NMR spectra analysis of target compound **3c** is shown in Figure 2.

For the ^13^C NMR spectrum, the signals at 180.21–109.04 ppm were assigned to the carbon of the aromatic ring, and the other peaks appearing at the range of 84.87–11.60 ppm were the signals of steroidal carbons. All mass spectra were obtained on a Waters ACQUITY UPLC^®^ H-CLASS PDA (Waters^®^) instrument. According to the experiments displayed, the ESI-MS spectrum of the target derivatives revealed a distinct molecular peak in positive ion mode, the value of [M + H]^+^ absorption signal was consistent with the calculated value of the molecular weight.

### Cytotoxic activity

#### The in vitro cytotoxic activity of target derivatives 3a–h and 6a–h

**Figure 3.**
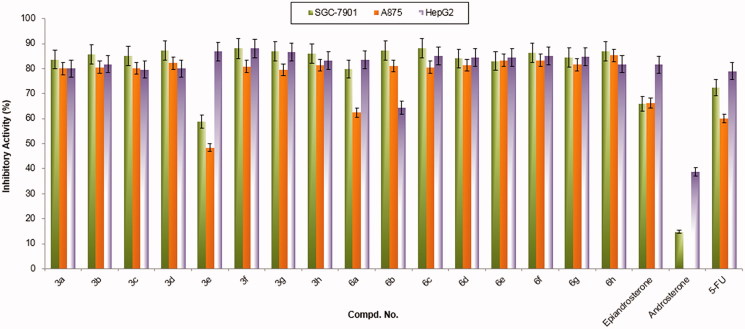
Antitumor activities of compounds **3a–h** and **6a–h** at 20 µg/mL. SGC-7901: Human gastric cancer cell line; A875: Human melanoma cell line; HepG2: Human hepatocellular liver carcinoma cell line; 5-FU: 5-Fluorouracil, used as a positive control.

All obtained compounds **3a–h** and **6a–h** were screened for their *in vitro* cytotoxic activity against SGC-7901 (Human gastric cancer), A875 (human melanoma), and HepG2 (human hepatocellular liver carcinoma) cell lines using the standard MTT assay^24–26^ compared with 5-FU (5-Fluorouracil). The preliminary cytotoxic screening results were summarized in Figure 3.

According to the results shown in Figure 3, most of these steroidal isatin derivatives **3a–h** and **6a–h** present good cytotoxic activities (75–85%) against the tested cell lines compared with the control at the concentration of 20 µg/mL. Notably, the androsterone-isatin conjugates **6a–h** exhibited better inhibitory activities against all tested cell lines than that of the parent compound androsterone. Meanwhile, the epiandrosterone-isatin hybrids **3a–h** also indicated better inhibitory actiities than that of the parent epiandrosterone especially for SGC7901 and A875 cell lines. The preliminary results demonstrated that the strategy combined steroid and isatin was effective, and these novel steroidal isatin hybrids might be used as a potential active scaffold for optimization of cytotoxic agents.

In addition, the IC_50_ values for all derivatives were also evaluated, and the cytotoxic activities expressed as IC_50_ values are presented in the following Table 1. From these data, we can find most of the derivatives exhibited higher inhibition activity than the commercial 5-FU, and the activity of epiandrosterone-isatin derivatives **3a–h** are slightly better than that of androsterone-isatin hybrids **6a–h**. Especially, the compounds with 5-I group have poor activities (Entries 5 and 13), and the two parent compounds epiandrosterone (Entry 17) and androsterone (Entry 18) also indicated poor cytotoxic activity at the same conditions. These results can further confirm the cytotoxic activity of these steroidal isatin derivatives are highly potential scaffold to discovery of potential anti-cancer agents. Meanwhile, the selectivity of the target derivatives on tumor cell lines and normal cells have been investigated and the non-cancer cells HL-7702 have been used in our experiment, and the results in Table 1 indicated that most of the obtained compounds exhibited cytotoxic activity against HL-7702 cell lines, and the cytotoxic activities of compound **6g** exhibited some selective cytotoxicity between cancer cells (IC_50_ 3.85–6.40 μM) and normal cells (HL-7702, IC_50_ = 11.23 μM). In addition, this class of steroidal isatin derivatives **3a–h** and **6a–h** exhibited slightly better inhibitory activities than that of the steroidal derivatives derived from dehydroepiandrosterone^19^^,^^20^, which deserved to carry out further structural optimization and in-depth study.

**Table 1.** Cytotoxic activity of the steroid-isatin hybrids.

Entry	Compd. No.	Substituents (R)	In vitro cytotoxicity IC_50_^a^ (μM)			
			SGC-7901	A875	HepG2	HL-7702
1	**3a**	H	7.69 ± 2.54	9.51 ± 1.34	6.72 ± 1.59	5.65 ± 1.59
2	**3b**	5-F	6.67 ± 1.77	8.58 ± 1.64	6.12 ± 1.79	5.49 ± 1.33
3	**3c**	5-Cl	4.92 ± 1.13	7.92 ± 1.95	2.39 ± 0.36	5.01 ± 1.37
4	**3d**	5-Br	8.43 ± 2.23	11.58 ± 2.84	8.29 ± 2.41	14.06 ± 3.79
5	**3e**	5-I	21.67 ± 3.86	23.75 ± 4.39	18.62 ± 4.79	13.95 ± 2.83
6	**3f**	5-CH_3_	7.06 ± 1.27	6.01 ± 1.95	6.91 ± 0.58	6.08 ± 0.54
7	**3g**	5-NO_2_	11.88 ± 2.45	26.20 ± 4.59	19.44 ± 3.37	33.39 ± 6.15
8	**3h**	6-F	7.22 ± 1.17	6.85 ± 1.99	4.85 ± 0.84	7.87 ± 2.04
9	**6a**	H	13.82 ± 2.65	21.72 ± 4.82	20.36 ± 6.69	8.29 ± 2.24
10	**6b**	5-F	9.51 ± 0.49	42.21 ± 8.19	32.31 ± 4.21	7.09 ± 1.57
11	**6c**	5-Cl	6.83 ± 2.01	8.78 ± 0.64	4.84 ± 1.05	6.36 ± 1.37
12	**6d**	5-Br	7.14 ± 1.41	8.08 ± 0.51	6.10 ± 2.09	4.40 ± 0.68
13	**6e**	5-I	17.11 ± 3.68	19.72 ± 1.89	8.99 ± 3.13	18.49 ± 1.00
14	**6f**	5-CH_3_	9.12 ± 1.61	8.25 ± 1.97	4.85 ± 1.07	12.50 ± 4.36
15	**6g**	5-NO_2_	6.40 ± 1.74	3.85 ± 1.44	4.31 ± 0.92	11.23 ± 3.05
16	**6h**	6-F	9.20 ± 1.68	7.71 ± 2.13	5.41 ± 1.44	12.76 ± 2.79
17	**Epiandrosterone**	**–**	69.77 ± 8.47	63.57 ± 13.22	36.67 ± 10.61	91.24 ± 12.29
18	**Androsterone**	**–**	>100	>100	>100	>100
19	**5-FU**^b^	**–**	62.03 ± 8.38	65.26 ± 7.30	63.18 ± 12.61	86.02 ± 9.61

Abbreviations: SGC-7901: Human gastric cancer cell line; A875: Human melanoma cell line; HepG2: Human hepatocellular liver carcinoma cell line; HL-7702: Human normal liver cells.

a IC_50_: Compound concentration required to inhibit tumor cell proliferation by 50%.

b 5-Fluorouracil, used as a positive control.

**Figure 4.**
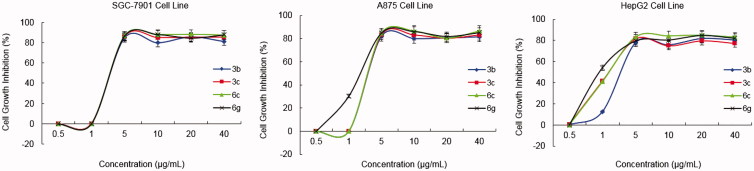
Dose-response analysis of cell growth inhibition activity for representative compounds **3b**, **3c**, **6c** and **6g** against SGC-7901 (left), A875 (middle) and HepG2 (right) cell lines.

The Figure 4 described the dose-response curve of cell growth inhibition activities for high potential compounds **3b**, **3c**, **6c** and **6g**, which indicated that these steroidal isatin derivatives exhibited cytotoxic effects on SGC-7901, A875 and HepG2 cell lines in a dose-dependent manner.

#### The structure and activity relationships (SARs)

According to the *in vitro* bioassay results presented in the Figure 3 and Table 1, the possible structure-activity relationships for these steroidal isatin derivatives can be obtained.

First, we can observe that most of these conjugates exhibited higher activity than those of parent compounds, and the compounds from the epiandrosterone series **3a–h** were slightly better than those from androsterone series **6a–h**. For the epiandrosterone series, the 5-I or 5-NO_2_ group decrease the cytotoxicity of the corresponding compounds (**3e** or **3g**), which may be due to the large size of these groups is unfavorable for activity. The compound **3c** with 5-Cl substituent is the highest potent against tested cell lines. For the androsterone series, the 5-I group also decrease the potential cytotoxicity of the corresponding compound (**6e**), and the range of IC_50_ value is 8–20 µM. Especially, the compound bearing 5-F substituent **6b** present a wide IC_50_ range, which exhibit selective inhibition against SGC-7901 cell lines (IC_50_ 9.51 ± 0.49 µM). In particular, it’s interesting that the two compounds containing a nitro-group (**3g** and **6g**) present significant activity difference, which testified the configuration might also affect the activity. According to these findings, it can be speculated it would have been more interesting to test the further activity of the nitro-group derivatives against other cell lines, and the special properties of different configuration will be helpful to investigate the real potent.

## Conclusion

Two series of steroidal isatin derivatives were synthesized, characterized and evaluated for their *in vitro* cytotoxicity against three cancer cell lines (SGC-7901, A875, HepG2). The preliminary bioassay indicated that most of the newly synthesized compounds exhibited good cytotoxic activities against tested cell lines compared with 5-fluorouracil (5-FU), especially, the two compounds containing a nitro-group (**3g** and **6g**) present significant activity difference. These findings will help to design and discover novel anticancer agents.

## Experimental section

### Instrumentation and chemicals

All starting materials and reagents commercially available were used without further purification, unless otherwise specified. ^1^H NMR and ^13^C NMR spectra were recorded on a Bruker Avance III 600 MHz FT-NMR spectrometer using DMSO-d_6_ as the solvent and tetramethylsilane (TMS) as the internal standard. Chemical shifts are reported in *δ* (parts per million) values, and coupling constants ^n^*J* are reported in Hz. Mass spectra were performed on a Waters ACQUITY UPLC^®^ H-CLASS PDA (Waters^®^) instrument. Analytical thin-layer chromatography was carried out on precoated silica gel plates GF254 (Qindao Haiyang Chemical, China), and spots were visualized with ultraviolet light.

### General synthetic procedure for target compounds

The key intermediates (Compd. **2** and **5**) and target steroidal derivatives **3a–h** and **6a–h** were conveniently synthesized according to the modified procedures^20^. The general process for preparation of these steroidal isatin derivatives is shown as following: To a solution of intermediates **2** or **5** (2 mmol) in ethanol (20 ml) was added the equal amounts of substituted isatin (2 mmol), and then the mixture was stirred at 30–45 °C for additional hours. After that, the solution was concentrated, and the condensation product separated out on cooling (0–4 °C) and was recrystallized from ethanol. All the compounds were characterized by ESI-MS, ^1^H NMR and ^13^C NMR spectroscopic analyses. Their physico-chemical properties and spectra data are as follows:

#### 3-(((3S,8R,9S,10S,13S,14S)-3-Hydroxy-10,13-dimethyldodecahydro-1H-cyclopenta[a]phenanthren-17(2H,10H,14H)-ylidene)hydrazono)indolin-2-one 3a

This compound was obtained following the above method as bright yellow powder, yield 73%. ^1^H NMR (600 MHz, DMSO-d_6_): *δ* = 10.77 (s, 1H), 7.79 (d, *J* = 7.6 Hz, 1H), 7.37–7.35 (m, 1H), 7.01 (t, *J* = 7.6 Hz, 1H), 6.87 (d, *J* = 7.8 Hz, 1H), 4.44 (d, *J* = 4.8 Hz, 1H), 3.39–3.34 (m, 1H), 2.39 (dd, *J* = 19.3, 8.4 Hz, 1H), 2.32–2.21 (m, 1H), 2.03 (d, *J* = 12.4 Hz, 1H), 1.84–1.73 (m, 1H), 1.71–1.59 (m, 4H), 1.55–1.40 (m, 3H), 1.39–1.31 (m, 2H), 1.19 (s, 4H), 1.15 (s, 1H), 1.06 (d, *J* = 12.3 Hz, 1H), 1.01–0.89 (m, 5H), 0.80 (s, 3H), 0.77–0.70 (m, 1H); ^13^C NMR (150 MHz, DMSO-d_6_): *δ* = 178.12, 164.73, 146.78, 144.91, 133.54, 128.53, 122.54, 117.11, 111.11, 69.75, 54.38, 53.10, 45.20, 44.85, 38.63, 37.07, 35.78, 35.03, 34.21, 31.84, 31.51, 28.71, 27.96, 23.31, 20.89, 16.97, 12.60; MS (ESI) *m/z* 434.5 (M + H)^+^, calcd. for C_27_H_35_N_3_O_2_ *m/z* = 433.3; HRMS: calcd for C_27_H_35_N_3_O_2_ ([M + H]^+^), 434.5857; found, 434.6535.

#### 5-Fluoro-3-(((3S,8R,9S,10S,13S,14S)-3-hydroxy-10,13-dimethyldodecahydro-1H-cyclopenta[a]phenanthren-17(2H,10H,14H)-ylidene)hydrazono)indolin-2-one 3b

This compound was obtained following the above method as brown yellow powder, yield 65%. ^1^H NMR (600 MHz, DMSO-d_6_): *δ* = 10.82 (s, 1H), 7.56 (dd, *J* = 8.4, 2.7 Hz, 1H), 7.27–7.23 (m, 1H), 6.88 (dd, *J* = 8.6, 4.3 Hz, 1H), 4.44 (d, *J* = 4.7 Hz, 1H), 3.41–3.33 (m, 1H), 2.45 (dd, *J* = 19.4, 8.6 Hz, 1H), 2.37–2.26 (m, 1H), 2.02 (d, *J* = 12.2 Hz, 1H), 1.81–1.74 (m, 1H), 1.74–1.60 (m, 4H), 1.55–1.32 (m, 5H), 1.31–1.13 (m, 5H), 1.09–1.03 (m, 1H), 0.99–0.88 (m, 5H), 0.80 (s, 3H), 0.76–0.69 (m, 1H); ^13^C NMR (150 MHz, DMSO-d_6_): *δ* = 180.14, 164.74, 156.98, 146.98, 141.32, 120.01, 119.85, 117.50, 115.35, 112.18, 69.74, 54.38, 53.06, 45.36, 44.84, 38.62, 37.06, 35.77, 35.01, 34.21, 31.84, 31.50, 28.70, 28.15, 23.30, 20.89, 17.00, 12.58; MS (ESI) *m/z* 452.5 (M + H)^+^, calcd. for C_27_H_34_FN_3_O_2_ *m/z* = 451.3; HRMS: calcd for C_27_H_34_FN_3_O_2_ ([M + H]^+^), 452.5762; found, 452.6553.

#### 5-Chloro-3-(((3S,8R,9S,10S,13S,14S)-3-hydroxy-10,13-dimethyldodecahydro-1H-cyclopenta[a]phenanthren-17(2H,10H,14H)-ylidene)hydrazono)indolin-2-one 3c

This compound was obtained following the above method as bright yellow powder, yield 72%. ^1^H NMR (600 MHz, DMSO-d_6_): *δ* = 10.93 (s, 1H), 7.81 (d, *J* = 2.2 Hz, 1H), 7.43 (dd, *J* = 8.3, 2.2 Hz, 1H), 6.90 (d, *J* = 8.4 Hz, 1H), 4.44 (d, *J* = 4.7 Hz, 1H), 3.41–3.34 (m, 1H), 2.45–2.27 (m, 2H), 2.01 (d, *J* = 12 Hz, 1H), 1.80–1.60 (m, 5H), 1.55–1.33 (m, 5H), 1.31–1.11 (m, 5H), 1.11–1.02 (m, 1H), 1.00–0.88 (m, 5H), 0.80 (s, 3H), 0.72 (t, *J* = 11.2 Hz, 1H); ^13^C NMR (150 MHz, DMSO-d_6_): *δ* = 179.58, 164.42, 146.30, 143.66, 132.93, 127.99, 126.02, 118.24, 112.71, 69.74, 54.42, 53.04, 45.37, 44.84, 38.61, 37.07, 35.77, 35.00, 34.24, 31.83, 31.49, 28.70, 28.11, 23.31, 20.93, 16.99, 12.56; MS (ESI) *m/z* 468.4 (M + H)^+^, calcd. for C_27_H_34_ClN_3_O_2_ *m/z* = 467.2; HRMS: calcd for C_27_H_34_ClN_3_O_2_ ([M + H]^+^), 469.0308; found, 468.5869.

#### 5-Bromo-3-(((3S,8R,9S,10S,13S,14S)-3-hydroxy-10,13-dimethyldodecahydro-1H-cyclopenta[a]phenanthren-17(2H,10H,14H)-ylidene)hydrazono)indolin-2-one 3d

This compound was obtained following the above method as yellow powder, yield 81%. ^1^H NMR (600 MHz, DMSO-d_6_): *δ* = 10.93 (s, 1H), 7.95 (d, *J* = 1.9 Hz, 1H), 7.55 (dd, *J* = 8.3, 2.1 Hz, 1H), 6.85 (d, *J* = 8.3 Hz, 1H), 4.44 (d, *J* = 4.6 Hz, 1H), 3.46–3.43 (m, 1H), 2.46–2.38 (m, 1H), 2.34–2.25 (m, 1H), 2.00 (d, *J* = 9.6 Hz, 1H), 1.81–1.62 (m, 5H), 1.53–1.35 (m, 5H), 1.28–1.05 (m, 6H), 1.00–0.90 (m, 5H), 0.81 (s, 3H), 0.77 (s, 1H); ^13^C NMR (150 MHz, DMSO-d_6_): *δ* = 179.18, 164.50, 145.10, 143.96, 135.89, 130.32, 125.50, 118.91, 113.60, 69.90, 54.58, 53.14, 45.55, 44.90, 38.62, 37.20, 35.75, 35.08, 34.31, 32.95, 31.55, 28.81, 28.02, 23.44, 20.95, 17.03, 12.59; MS (ESI) *m/z* 512.3 (M + H)^+^, calcd. for C_27_H_34_BrN_3_O_2_ *m/z* = 511.2; HRMS: calcd for C_27_H_34_BrN_3_O_2_ ([M + H]^+^), 512.1834; found, 512.5112.

#### 3-(((3S,8R,9S,10S,13S,14S)-3-Hydroxy-10,13-dimethyldodecahydro-1H-cyclopenta[a]phenanthren-17(2H,10H,14H)-ylidene)hydrazono)-5-iodoindolin-2-one 3e

This compound was obtained following the above method as yellow powder, yield 74%. ^1^H NMR (600 MHz, DMSO-d_6_): *δ* = 10.99 (s, 1H), 8.15 (d, *J* = 1.7 Hz, 1H), 7.68 (dd, *J* = 8.2, 1.8 Hz, 1H), 6.76 (d, *J* = 8.2 Hz, 1H), 4.46 (d, *J* = 4.7 Hz, 1H), 3.38–3.32 (m, 1H), 2.42–2.21 (m, 2H), 2.00 (d, *J* = 12.3 Hz, 1H), 1.82–1.73 (m, 1H), 1.73–1.60 (m, 4H), 1.55–1.34 (m, 5H), 1.23 (m, 4H), 1.18–1.12 (m, 1H), 1.05 (t, *J* = 5.7 Hz, 1H), 1.01–0.89 (m, 5H), 0.80 (s, 3H), 0.77–0.71 (m, 1H); ^13^C NMR (150 MHz, DMSO-d_6_): *δ* = 178.15, 164.02, 145.66, 144.37, 141.27, 136.48, 119.14, 113.60, 84.87, 69.72, 54.48, 53.06, 45.37, 44.84, 38.61, 37.11, 35.77, 34.99, 34.25, 31.84, 31.50, 28.70, 27.96, 23.31, 20.99, 16.94, 12.56; MS (ESI) *m/z* 560.3 (M + H)^+^, calcd. for C_27_H_34_IN_3_O_2_ *m/z* = 559.2.

#### 3-(((3S,8R,9S,10S,13S,14S)-3-Hydroxy-10,13-dimethyldodecahydro-1H-cyclopenta[a]phenanthren-17(2H,10H,14H)-ylidene)hydrazono)-5-methylindolin-2-one 3f

This compound was obtained following the above method as yellow powder, yield 68%. ^1^H NMR (600 MHz, DMSO-d_6_): *δ* = 10.67 (s, 1H), 7.62 (s, 1H), 7.18 (d, *J* = 8.7 Hz, 1H), 6.77 (d, *J* = 7.9 Hz, 1H), 4.44 (d, *J* = 4.7 Hz, 1H), 3.41–3.35 (m, 1H), 2.40–2.24 (m, 2H), 2.22 (s, 3H), 2.04 (d, *J* = 9.6 Hz, 1H), 1.75–1.40 (m, 10H), 1.30–1.11 (m, 5H), 1.10–1.03 (m, 1H), 1.03–0.88 (m, 5H), 0.80 (s, 3H), 0.77–0.70 (m, 1H); ^13^C NMR (150 MHz, DMSO-d_6_): *δ* = 177.12, 164.78, 146.60, 142.57, 133.77, 131.22, 128.99, 117.15, 110.88, 69.75, 54.45, 53.10, 45.18, 44.85, 38.62, 37.10, 35.78, 35.01, 34.26, 31.84, 31.51, 28.71, 27.88, 23.33, 21.14, 20.96, 16.92, 12.58; MS (ESI) *m/z* 448.4 (M + H)^+^, calcd. for C_28_H_37_N_3_O_2_ *m/z* = 447.3.

#### 3-(((3S,8R,9S,10S,13S,14S)-3-Hydroxy-10,13-dimethyldodecahydro-1H-cyclopenta[a]phenanthren-17(2H,10H,14H)-ylidene)hydrazono)-5-nitroindolin-2-one 3g

This compound was obtained following the above method as bright yellow powder, yield 78%. ^1^H NMR (600 MHz, DMSO-d_6_): *δ* = 11.50 (s, 1H), 8.65 (d, *J* = 2.4 Hz, 1H), 8.30 (dd, *J* = 8.7, 2.4 Hz, 1H), 7.07 (d, *J* = 8.7 Hz, 1H), 4.44 (d, *J* = 4.7 Hz, 1H), 3.38–3.34 (m, 1H), 2.47–2.27 (m, 2H), 2.06 (d, *J* = 12.6 Hz, 1H), 1.80–1.35 (m, 10H), 1.32–1.12 (m, 5H), 1.06 (d, *J* = 7.0 Hz, 1H), 1.03–0.88 (m, 5H), 0.82 (s, 3H), 0.75 (t, *J* = 11.4 Hz, 1H); ^13^C NMR (150 MHz, DMSO-d_6_): *δ* = 179.37, 164.91, 150.35, 145.44, 142.44, 129.69, 123.68, 116.90, 111.39, 69.74, 54.45, 53.04, 45.44, 44.86, 38.61, 37.11, 35.78, 35.01, 34.20, 31.85, 31.50, 28.69, 28.16, 23.32, 20.99, 16.96, 12.58; MS (ESI) *m/z* 479.4 (M + H)^+^, calcd. for C_27_H_34_N_4_O_4_ *m/z* = 478.3.

#### 6-Fluoro-3-(((3S,8R,9S,10S,13S,14S)-3-hydroxy-10,13-dimethyldodecahydro-1H-cyclopenta[a]phenanthren-17(2H,10H,14H)-ylidene)hydrazono)indolin-2-one 3h

This compound was obtained following the above method as brown yellow powder, yield 62%. ^1^H NMR (600 MHz, DMSO-d_6_): *δ* = 10.94 (s, 1H), 7.84 (dd, *J* = 8.4, 5.9 Hz, 1H), 6.87–6.82 (m, 1H), 6.70 (dd, *J* = 9.1, 2.3 Hz, 1H), 4.44 (d, *J* = 4.7 Hz, 1H), 3.38–3.35 (m, 1H), 2.45–2.26 (m, 2H), 2.01 (d, *J* = 9.4 Hz, 1H), 1.80–1.73 (m, 1H), 1.70–1.60 (m, 4H), 1.54–1.34 (m, 5H), 1.30–1.25 (m, 4H), 1.18–1.12 (m, 1H), 1.06 (t, *J* = 7.0 Hz, 1H), 0.98–0.90 (m, 5H), 0.80 (s, 3H), 0.73 (d, *J* = 9.3 Hz, 1H); ^13^C NMR (150 MHz, DMSO-d_6_): *δ* = 179.51, 165.05, 147.23, 146.04, 130.85, 130.77, 113.91, 109.20, 109.04, 69.75, 54.37, 53.06, 45.26, 44.85, 38.62, 37.06, 35.77, 35.03, 34.17, 31.83, 31.51, 28.70, 28.05, 23.30, 20.88, 16.99, 12.59; MS (ESI) *m/z* 452.4 (M + H)^+^, calcd. for C_27_H_34_FN_3_O_2_ *m/z* = 451.3.

#### 3-(((3R,5S,8R,9S,10S,13S,14S)-3-Hydroxy-10,13-dimethyldodecahydro-1H-cyclopenta[a]phenanthren-17(2H,10H,14H)-ylidene)hydrazono)indolin-2-one 6a

This compound was obtained following the above method as bright yellow powder, yield 77%. ^1^H NMR (600 MHz, DMSO-d_6_): *δ* = 10.77 (s, 1H), 7.80 (d, *J* = 7.2 Hz, 1H), 7.38–7.35 (m, 1H), 7.03–7.00 (m, 1H), 6.87 (d, *J* = 7.8 Hz, 1H), 4.18 (d, *J* = 3.0 Hz, 1H), 3.82 (d, *J* = 2.6 Hz, 1H), 2.42–2.22 (m, 2H), 2.03 (dd, *J* = 9.5, 3.0 Hz, 1H), 1.81–1.65 (m, 3H), 1.63–1.44 (m, 5H), 1.43–1.28 (m, 5H), 1.25–1.12 (m, 4H), 1.01–0.92 (m, 4H), 0.86–0.75 (m, 4H); ^13^C NMR (150 MHz, DMSO-d_6_): *δ* = 178.18, 164.74, 146.78, 144.91, 133.53, 128.54, 122.54, 117.11, 111.11, 64.53, 54.56, 53.18, 45.21, 39.03, 36.30, 36.20, 35.04, 34.23, 32.40, 31.63, 29.12, 28.59, 27.96, 23.28, 20.46, 16.98, 11.64; MS (ESI) *m/z* 434.5 (M + H)^+^, calcd. for C_27_H_35_N_3_O_2_ *m/z* = 433.3; HRMS: calcd for C_27_H_35_N_3_O_2_ ([M + H]^+^), 434.5857; found, 434.5635.

#### 5-Fluoro-3-(((3R,5S,8R,9S,10S,13S,14S)-3-hydroxy-10,13-dimethyldodecahydro-1H-cyclopenta[a]phenanthren-17(2H,10H,14H)-ylidene)hydrazono)indolin-2-one 6b

This compound was obtained following the above method as yellow powder, yield 71%. ^1^H NMR (600 MHz, DMSO-d_6_): *δ* = 10.82 (s, 1H), 7.57 (dd, *J* = 8.4, 2.7 Hz, 1H), 7.27–7.24 (m, 1H), 6.89 (dd, *J* = 8.6, 4.3 Hz, 1H), 4.19 (d, *J* = 3.0 Hz, 1H), 3.82 (d, *J* = 2.6 Hz, 1H), 2.48–2.29 (m, 2H), 2.03 (d, *J* = 12.4 Hz, 1H), 1.82–1.65 (m, 3H), 1.60–1.42 (m, 5H), 1.40–1.30 (m, 5H), 1.28–1.13 (m, 4H), 1.07–0.89 (m, 4H), 0.87–0.74 (m, 4H); ^13^C NMR (150 MHz, DMSO-d_6_): *δ* = 180.21, 164.75, 158.55, 156.98, 146.98, 141.30, 119.84, 117.53, 112.18, 64.53, 54.56, 53.12, 45.37, 39.03, 36.30, 36.20, 35.03, 34.23, 32.40, 31.62, 29.11, 28.58, 28.14, 23.27, 20.46, 17.01, 11.62; MS (ESI) *m/z* 452.5 (M + H)^+^, calcd. for C_27_H_34_FN_3_O_2_ *m/z* = 451.3; HRMS: calcd for C_27_H_34_FN_3_O_2_ ([M + H]^+^), 452.5762; found, 452.6553.

#### 5-Chloro-3-(((3R,5S,8R,9S,10S,13S,14S)-3-hydroxy-10,13-dimethyldodecahydro-1H-cyclopenta[a]phenanthren-17(2H,10H,14H)-ylidene)hydrazono)indolin-2-one 6c

This compound was obtained following the above method as deep yellow powder, yield 75%. ^1^H NMR (600 MHz, DMSO-d_6_): *δ* = 10.93 (s, 1H), 7.82 (d, *J* = 2.2 Hz, 1H), 7.43 (dd, *J* = 8.4, 2.3 Hz, 1H), 6.90 (d, *J* = 8.4 Hz, 1H), 4.19 (d, *J* = 3.0 Hz, 1H), 3.82 (d, *J* = 2.6 Hz, 1H), 2.45–2.23 (m, 2H), 2.02 (d, *J* = 12.4 Hz, 1H), 1.82–1.66 (m, 3H), 1.61–1.44 (m, 5H), 1.43–1.28 (m, 5H), 1.26–1.10 (m, 4H), 1.03–0.92 (m, 4H), 0.85–0.75 (m, 4H); ^13^C NMR (150 MHz, DMSO-d_6_): *δ* = 179.48, 164.42, 146.24, 143.65, 132.91, 127.98, 126.02, 118.24, 112.69, 64.53, 54.61, 53.12, 45.37, 39.02, 36.29, 36.19, 35.01, 34.25, 32.41, 31.62, 29.11, 28.58, 28.09, 23.28, 20.50, 16.99, 11.60; MS (ESI) *m/z* 468.4 (M + H)^+^, calcd. for C_27_H_34_ClN_3_O_2_ *m/z* = 467.2; HRMS: calcd for C_27_H_34_ClN_3_O_2_ ([M + H]^+^), 469.0308; found, 468.6769.

#### 5-Bromo-3-(((3R,5S,8R,9S,10S,13S,14S)-3-hydroxy-10,13-dimethyldodecahydro-1H-cyclopenta[a]phenanthren-17(2H,10H,14H)-ylidene)hydrazono)indolin-2-one 6d

This compound was obtained following the above method as yellowish powder, yield 84%. ^1^H NMR (600 MHz, DMSO-d_6_): *δ* = 10.93 (s, 1H), 7.96 (d, *J* = 2.0 Hz, 1H), 7.55 (dd, *J* = 8.3, 2.1 Hz, 1H), 6.85 (d, *J* = 8.3 Hz, 1H), 4.21 (d, *J* = 8.8 Hz, 1H), 3.86–3.78 (m, 1H), 2.45–2.24 (m, 2H), 2.01 (d, *J* = 12.3 Hz, 1H), 1.82–1.66 (m, 3H), 1.61–1.43 (m, 5H), 1.40–1.25 (m, 7H), 1.21–1.12 (m, 2H), 1.03–0.91 (m, 4H), 0.82–0.74 (m, 4H); ^13^C NMR (150 MHz, DMSO-d_6_): *δ* = 178.96, 164.27, 145.94, 143.99, 135.67, 130.74, 118.70, 113.60, 113.18, 64.53, 54.64, 53.14, 45.38, 39.03, 36.30, 36.19, 35.01, 34.27, 32.43, 31.63, 29.12, 28.58, 28.05, 23.28, 20.52, 16.99, 11.62; MS (ESI) *m/z* 512.4 (M + H)^+^, calcd. for C_27_H_34_BrN_3_O_2_ *m/z* = 511.2; HRMS: calcd for C_27_H_34_BrN_3_O_2_ ([M + H]^+^), 512.1834; found, 512.5112.

#### 3-(((3R,5S,8R,9S,10S,13S,14S)-3-Hydroxy-10,13-dimethyldodecahydro-1H-cyclopenta[a]phenanthren-17(2H,10H,14H)-ylidene)hydrazono)-5-iodoindolin-2-one 6e

This compound was obtained following the above method as yellow powder, yield 79%. ^1^H NMR (600 MHz, DMSO-d_6_): *δ* = 10.99 (s, 1H), 8.15 (d, *J* = 6.0 Hz, 1H), 7.69 (dd, *J* = 6.0 Hz, 1H), 6.76 (d, *J* = 8.4 Hz, 1H), 4.22 (d, *J* = 6.0 Hz, 1H), 3.85–3.79 (m, 1H), 2.41–2.23 (m, 2H), 2.02–1.98 (m, 1H), 1.80–1.75 (m, 1H), 1.72–1.70 (m, 2H), 1.59–1.45 (m, 5H), 1.39–1.30 (m, 4H), 1.28–1.22 (m, 5H), 1.19–1.12 (m, 2H), 0.99 (s, 3H), 0.79 (s, 3H); ^13^C NMR (150 MHz, DMSO-d_6_): *δ* = 177.88, 164.01, 144.37, 141.26, 136.42, 129.17, 123.47, 119.14, 113.61, 64.52, 54.67, 53.17, 45.37, 43.67, 39.04, 36.31, 36.19, 35.00, 34.26, 32.44, 31.63, 28.58, 27.95, 23.28, 20.55, 16.95, 11.62; MS (ESI) *m/z* 560.2 (M + H)^+^, calcd. for C_27_H_34_IN_3_O_2_ *m/z* = 559.2.

#### 3-(((3R,5S,8R,9S,10S,13S,14S)-3-Hydroxy-10,13-dimethyldodecahydro-1H-cyclopenta[a]phenanthren-17(2H,10H,14H)-ylidene)hydrazono)-5-methylindolin-2-one 6f

This compound was obtained following the above method as yellow powder, yield 74%. ^1^H NMR (600 MHz, DMSO-d_6_): *δ* = 10.67 (s, 1H), 7.63 (s, 1H), 7.19 (dd, *J* = 6.0 Hz, 1H), 6.77 (d, *J* = 7.8 Hz, 1H), 3.82–3.80 (m, 2H), 2.39–2.26 (m, 2H), 2.23 (s, 3H), 1.72–1.69 (m, 2H), 1.53–1.45 (m, 5H), 1.38–1.32 (m, 6H), 1.28–1.22 (m, 4H), 1.18–1.12 (m, 3H), 0.98 (s, 3H), 0.79 (s, 3H); ^13^C NMR (150 MHz, DMSO-d_6_): *δ* = 177.01, 164.78, 146.53, 142.57, 133.75, 131.22, 128.98, 117.15, 110.88, 64.53, 54.64, 53.19, 45.18, 39.03, 36.31, 36.20, 35.02, 34.28, 32.44, 31.64, 29.12, 28.60, 27.86, 23.30, 21.14, 20.52, 16.93, 11.62; MS (ESI) *m/z* 448.4 (M + H)^+^, calcd. for C_28_H_37_N_3_O_2_ *m/z* = 447.3.

#### 3-(((3R,5S,8R,9S,10S,13S,14S)-3-Hydroxy-10,13-dimethyldodecahydro-1H-cyclopenta[a]phenanthren-17(2H,10H,14H)-ylidene)hydrazono)-5-nitroindolin-2-one 6g

This compound was obtained following the above method as bright yellow powder, yield 78%. ^1^H NMR (600 MHz, DMSO-d_6_): *δ* = 11.49 (s, 1H), 8.65 (d, *J* = 2.0 Hz, 1H), 8.30 (dd, *J* = 8.6, 2.1 Hz, 1H), 7.06 (d, *J* = 8.7 Hz, 1H), 4.19 (d, *J* = 1.9 Hz, 1H), 3.82 (s, 1H), 2.48–2.24 (m, 2H), 2.06 (d, *J* = 12.2 Hz, 1H), 1.85–1.65 (m, 3H), 1.66–1.46 (m, 5H), 1.42–1.13 (m, 9H), 1.08–0.93 (m, 4H), 0.90–0.66 (m, 4H); ^13^C NMR (150 MHz, DMSO-d_6_): *δ* = 179.37, 164.90, 150.33, 145.40, 142.43, 129.66, 123.67, 116.89, 111.37, 64.53, 54.61, 53.11, 45.44, 39.03, 36.30, 36.20, 35.02, 34.21, 32.43, 31.62, 29.13, 28.58, 28.14, 23.29, 20.56, 16.96, 11.61; MS (ESI) *m/z* 479.4 (M + H)^+^, calcd. for C_27_H_34_N_4_O_4_ *m/z* = 478.3.

#### 6-Fluoro-3-(((3R,5S,8R,9S,10S,13S,14S)-3-hydroxy-10,13-dimethyldodecahydro-1H-cyclopenta[a]phenanthren-17(2H,10H,14H)-ylidene)hydrazono)indolin-2-one 6h

This compound was obtained following the above method as brown yellow powder, yield 68%. ^1^H NMR (600 MHz, DMSO-d_6_): *δ* = 10.95 (s, 1H), 7.85 (dd, *J* = 8.4 Hz, 1H), 6.96–6.81 (m, 1H), 6.70 (dd, *J* = 9.1 Hz, 1H), 4.19 (d, *J* = 3.0 Hz, 1H), 3.85–3.80 (m, 1H), 2.45–2.25 (m, 2H), 2.03 (d, *J* = 9.5 Hz, 1H), 1.83–1.66 (m, 4H), 1.62–1.43 (m, 6H), 1.38–1.25 (m, 5H), 1.18–1.14 (m, 2H), 1.02–0.90 (m, 4H), 0.79 (s, 3H), 0.76 (s, 1H); ^13^C NMR (150 MHz, DMSO-d_6_): *δ* = 179.52, 165.06, 147.22, 146.01, 133.34, 130.84, 113.91, 109.20, 109.05, 64.53, 54.54, 53.14, 45.27, 44.26, 39.04, 36.30, 36.20, 35.04, 34.19, 32.39, 31.62, 29.11, 28.04, 23.27, 20.44, 17.00, 11.63; MS (ESI) *m/z* 452.4 (M + H)^+^, calcd. for C_27_H_34_FN_3_O_2_ *m/z* = 451.3.

### In vitro cytotoxic activity

The *in vitro* cytotoxicity of the synthesized compounds against different cell lines (SGC-7901, A875, and HepG2) were measured with the MTT assay^24–26^. Briefly, SGC-7901, A875 and HepG2 cells were seeded at 2 × 104 cells per well in 96-well plates and grown to subconfluence. After removal of the growth medium, six serial dilutions of each tested compound in 200 µL test medium were added. Plates were incubated at 37 °C in a humidified atmosphere containing 5% CO_2_. After 72 h of exposure, the culture medium was removed and 30 µL of the MTT solution (5 mg/mL in PBS) was added to each well. The plate was further incubated for 4 h to allow MTT formazan formation. To dissolve the resulting MTT formazan, 50 µL of DMSO was added to each well, followed by thorough mixing with a microplate shaker. Absorbance at 570 nm was measured on a microplate reader (Thermo Scientific, MK3). All data were analyzed with SPSS 16.0 software, and the 50% inhibitory concentrations (IC_50_) of each compound for the different cell lines were determined. Assays were performed in triplicate on three independent experiments.
